# (2*E*)-2-Benzyl­idene-5,6-dimethoxy­indan-1-one

**DOI:** 10.1107/S1600536810035695

**Published:** 2010-09-11

**Authors:** Mohamed Ashraf Ali, Rusli Ismail, Soo Choon Tan, Chin Sing Yeap, Hoong-Kun Fun

**Affiliations:** a Institute for Research in Molecular Medicine, Universiti Sains Malaysia, 11800 USM, Penang, Malaysia; b X-ray Crystallography Unit, School of Physics, Universiti Sains Malaysia, 11800 USM, Penang, Malaysia

## Abstract

The mol­ecular structure of the title compound, C_18_H_16_O_3_, is roughly planar; the maximum deviation of the indanone ring system is 0.027 (1) Å and it makes a dihedral angle of 2.69 (3)° with the phenyl ring. The torsion angles between the two meth­oxy groups and the ­indanone ring are −14.67 (11) and −1.11 (12)°. In the crystal, mol­ecules are connected into a ribbon along the *a* axis *via* weak inter­molecular C—H⋯O hydrogen bonds. Weak inter­molecular C—H⋯π and π–π [centroid–centroid distance = 3.7086 (6) Å] inter­actions are also observed.

## Related literature

For general background to and the biological activity of chalcone derivatives, see: Boumendjel *et al.* (2009 ▶); D’Archivio *et al.* (2008 ▶); Dicarlo *et al.* (1999 ▶); Echeverria *et al.* (2009 ▶); Heidenreich *et al.* (2008 ▶); Katsori & Hadjipavlou-Latina (2009 ▶); Miranda *et al.* (1999 ▶); Nowakowska (2007 ▶); Shah *et al.* (2008 ▶); Syed *et al.* (2008 ▶). For the stability of the temperature controller used in the data collection, see: Cosier & Glazer (1986 ▶).
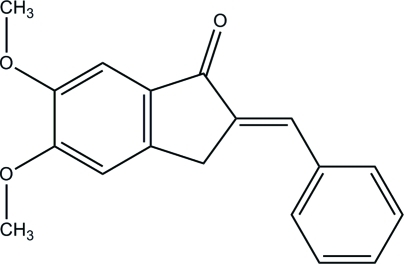


## Experimental

### 

#### Crystal data

C_18_H_16_O_3_
*M* *_r_* = 280.31
Monoclinic, 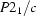

*a* = 6.0209 (6) Å
*b* = 14.8550 (14) Å
*c* = 15.2292 (15) Å
β = 90.603 (2)°
*V* = 1362.0 (2) Å^3^
*Z* = 4
Mo *K*α radiation
μ = 0.09 mm^−1^
*T* = 100 K
0.44 × 0.29 × 0.16 mm

#### Data collection

Bruker APEXII DUO CCD area-detector diffractometer
Absorption correction: multi-scan (*SADABS*; Bruker, 2009 ▶) *T*_min_ = 0.960, *T*_max_ = 0.985
31475 measured reflections
6003 independent reflections
4902 reflections with *I* > 2σ(*I*)
*R*_int_ = 0.048

#### Refinement

*R*[*F* ^2^ > 2σ(*F* ^2^)] = 0.043
*wR*(*F* ^2^) = 0.130
*S* = 1.09
6003 reflections
192 parameters
H-atom parameters constrained
Δρ_max_ = 0.60 e Å^−3^
Δρ_min_ = −0.58 e Å^−3^

### 

Data collection: *APEX2* (Bruker, 2009 ▶); cell refinement: *SAINT* (Bruker, 2009 ▶); data reduction: *SAINT*; program(s) used to solve structure: *SHELXTL* (Sheldrick, 2008 ▶); program(s) used to refine structure: *SHELXTL*; molecular graphics: *SHELXTL*; software used to prepare material for publication: *SHELXTL* and *PLATON* (Spek, 2009 ▶).

## Supplementary Material

Crystal structure: contains datablocks global, I. DOI: 10.1107/S1600536810035695/is2598sup1.cif

Structure factors: contains datablocks I. DOI: 10.1107/S1600536810035695/is2598Isup2.hkl

Additional supplementary materials:  crystallographic information; 3D view; checkCIF report

## Figures and Tables

**Table 1:** Hydrogen-bond geometry (Å, °) *Cg*1 is the centroid of C2–C7 benzene ring.

*D*—H⋯*A*	*D*—H	H⋯*A*	*D*⋯*A*	*D*—H⋯*A*
C17—H17*A*⋯O3^i^	0.96	2.53	3.4107 (11)	152
C18—H18*B*⋯O2^ii^	0.96	2.58	3.5320 (11)	173
C16—H16*A*⋯*Cg*1^iii^	0.93	2.99	3.7224 (9)	137

Symmetry codes: (i) 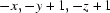; (ii) 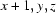; (iii) 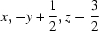.

**Fig. 1.**
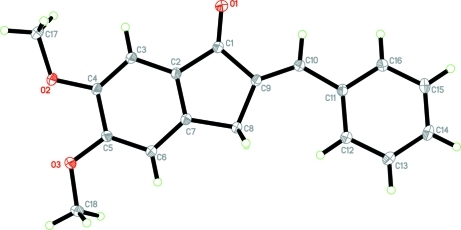
The molecular structure of the title compound with atom labels and 50% probability ellipsoids for non-H atoms.

**Fig. 2.**
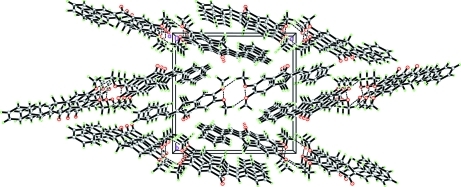
The crystal packing of title compound, showing chains along the [100] direction. Intermolecular hydrogen bonds are shown as dashed lines.

## supplementary crystallographic information

### Comment

Chalcones have been reported to possess antiinflammatory, antimicrobial, antioxidant and anticancer properties (Echeverria *et al.*, 2009; Nowakowska, 2007; Miranda *et al.*, 1999; Shah *et al.*, 2008; Boumendjel *et al.*, 2009; Katsori & Hadjipavlou-Latina, 2009). Chalcones are one of the major classes of natural products with widespread distribution in spices, tea, beer, fruits and vegetables. They have been recently subjects of great interest for their pharmacological activities (Dicarlo *et al.*, 1999). Prostate cancer is one of the most commonly diagnosed cancers in men and the second leading cause of cancer deaths in the European Union and United States of America (Heidenreich *et al.*, 2008). Many antitumor drugs have been developed for prostate cancer patients, but their intolerable systemic toxicity often limits their clinical use. Chemoprevention is one of the most promising approaches in prostate cancer research, in which natural or synthetic agents are used to prevent this malignant disease (Heidenreich *et al.*, 2008; Syed *et al.*, 2008; D'Archivio *et al.*, 2008).

The molecular structure of the title compound is essentially coplanar (Fig. 1). The maximum deviation of the indanone group is 0.027 (1) Å and it makes dihedral angle of 2.69 (3)° with the phenyl ring [C11–C16]. The torsion angles of the two methoxy groups are [C17–O2–C4–C5] 165.99 (7) and [C18–O3–C5–C4] 179.19 (7)°.

In the crystal structure, intermolecular C17—H17A···O3 hydrogen bonds (Table 1) link the molecules into dimers (Fig. 2). These dimers are interconnected into ribbons propagating along the [100] direction *via* intermolecular C18—H18B···O2 hydrogen bonds (Fig. 2, Table 1). Weak intermolecular C—H···π (Table 1) and π–π interactions are also observed. [*Cg*1···*Cg*2^iv^ of 3.7086 (6) Å; (iv) 1 - *x*, 1 - *y*, -*z. Cg*1 and *Cg*2 are the centroids of C2–C7 and C11–C16 benzene ring.]

### Experimental

A mixture of 5,6-dimethoxyindan-1-one (0.001 mmol) and benzaldehyde (0.001 mmol) were dissolved in methanol (10 ml) and 30% sodium hydroxide solution (5 ml) was added and stirred for 5 h. After completion of the reaction as evident from TLC, the mixture was poured into crushed ice then neutralized with concentrated HCl. The precipitated solid was filtered, washed with water and recrystallized from ethanol to reveal the title compound as light yellow crystals.

### Refinement

All H atoms were positioned geometrically (C—H = 0.93–0.97 Å) and refined using a riding model. A rotating-group model were applied for the methyl groups.

### Crystal data

**Table:**

C_18_H_16_O_3_	*F*(000) = 592
*M**_r_* = 280.31	*D*_x_ = 1.367 Mg m^−^^3^
Monoclinic, *P*2_1_/*c*	Mo *K*α radiation, λ = 0.71073 Å
Hall symbol: -P 2ybc	Cell parameters from 7296 reflections
*a* = 6.0209 (6) Å	θ = 2.7–34.9°
*b* = 14.8550 (14) Å	µ = 0.09 mm^−^^1^
*c* = 15.2292 (15) Å	*T* = 100 K
β = 90.603 (2)°	Yellow, colourless
*V* = 1362.0 (2) Å^3^	0.44 × 0.29 × 0.16 mm
*Z* = 4	

### Data collection

**Table:**

Bruker APEXII DUO CCD area-detector diffractometer	6003 independent reflections
Radiation source: fine-focus sealed tube	4902 reflections with *I* > 2σ(*I*)
graphite	*R*_int_ = 0.048
φ and ω scans	θ_max_ = 35.0°, θ_min_ = 1.9°
Absorption correction: multi-scan (*SADABS*; Bruker, 2009)	*h* = −9→9
*T*_min_ = 0.960, *T*_max_ = 0.985	*k* = −23→23
31475 measured reflections	*l* = −23→24

### Refinement

**Table:**

Refinement on *F*^2^	Primary atom site location: structure-invariant direct methods
Least-squares matrix: full	Secondary atom site location: difference Fourier map
*R*[*F*^2^ > 2σ(*F*^2^)] = 0.043	Hydrogen site location: inferred from neighbouring sites
*wR*(*F*^2^) = 0.130	H-atom parameters constrained
*S* = 1.09	*w* = 1/[σ^2^(*F*_o_^2^) + (0.0698*P*)^2^ + 0.2363*P*] where *P* = (*F*_o_^2^ + 2*F*_c_^2^)/3
6003 reflections	(Δ/σ)_max_ = 0.001
192 parameters	Δρ_max_ = 0.60 e Å^−^^3^
0 restraints	Δρ_min_ = −0.58 e Å^−^^3^

### Special details

**Table:**

Experimental. The crystal was placed in the cold stream of an Oxford Cryosystems Cobra open-flow nitrogen cryostat (Cosier & Glazer, 1986) operating at 100.0 (1) K.
Geometry. All e.s.d.'s (except the e.s.d. in the dihedral angle between two l.s. planes) are estimated using the full covariance matrix. The cell e.s.d.'s are taken into account individually in the estimation of e.s.d.'s in distances, angles and torsion angles; correlations between e.s.d.'s in cell parameters are only used when they are defined by crystal symmetry. An approximate (isotropic) treatment of cell e.s.d.'s is used for estimating e.s.d.'s involving l.s. planes.
Refinement. Refinement of *F*^2^ against ALL reflections. The weighted *R*-factor *wR* and goodness of fit *S* are based on *F*^2^, conventional *R*-factors *R* are based on *F*, with *F* set to zero for negative *F*^2^. The threshold expression of *F*^2^ > σ(*F*^2^) is used only for calculating *R*-factors(gt) *etc*. and is not relevant to the choice of reflections for refinement. *R*-factors based on *F*^2^ are statistically about twice as large as those based on *F*, and *R*- factors based on ALL data will be even larger.

### Fractional atomic coordinates and isotropic or equivalent isotropic displacement parameters (Å^2^)

**Table:**

	*x*	*y*	*z*	*U*_iso_*/*U*_eq_	
O1	0.07043 (10)	0.71482 (4)	0.09037 (4)	0.01685 (13)	
O2	0.07512 (10)	0.55593 (4)	0.41523 (4)	0.01527 (12)	
O3	0.42675 (10)	0.46101 (4)	0.40716 (4)	0.01623 (13)	
C1	0.22764 (13)	0.66575 (5)	0.11035 (5)	0.01189 (13)	
C2	0.26214 (13)	0.61731 (5)	0.19344 (5)	0.01122 (13)	
C3	0.12486 (13)	0.61613 (5)	0.26768 (5)	0.01208 (13)	
H3A	−0.0055	0.6497	0.2689	0.014*	
C4	0.18842 (13)	0.56395 (5)	0.33862 (5)	0.01188 (13)	
C5	0.38768 (13)	0.51154 (5)	0.33496 (5)	0.01213 (13)	
C6	0.52425 (13)	0.51522 (5)	0.26166 (5)	0.01263 (14)	
H6A	0.6561	0.4827	0.2602	0.015*	
C7	0.45911 (12)	0.56873 (5)	0.19043 (5)	0.01121 (13)	
C8	0.57760 (13)	0.58179 (5)	0.10412 (5)	0.01231 (13)	
H8A	0.5966	0.5249	0.0738	0.015*	
H8B	0.7219	0.6095	0.1131	0.015*	
C9	0.42250 (13)	0.64368 (5)	0.05349 (5)	0.01186 (13)	
C10	0.43407 (13)	0.67809 (5)	−0.02828 (5)	0.01274 (14)	
H10A	0.3154	0.7150	−0.0441	0.015*	
C11	0.60191 (13)	0.66724 (5)	−0.09638 (5)	0.01229 (13)	
C12	0.79718 (14)	0.61621 (6)	−0.08618 (5)	0.01469 (14)	
H12A	0.8259	0.5868	−0.0334	0.018*	
C13	0.94801 (14)	0.60945 (6)	−0.15463 (6)	0.01690 (15)	
H13A	1.0760	0.5751	−0.1472	0.020*	
C14	0.90917 (15)	0.65353 (6)	−0.23413 (6)	0.01682 (15)	
H14A	1.0106	0.6487	−0.2795	0.020*	
C15	0.71743 (15)	0.70489 (6)	−0.24506 (6)	0.01576 (15)	
H15A	0.6910	0.7349	−0.2977	0.019*	
C16	0.56526 (14)	0.71135 (5)	−0.17716 (5)	0.01416 (14)	
H16A	0.4370	0.7454	−0.1853	0.017*	
C17	−0.09525 (14)	0.62140 (6)	0.43137 (6)	0.01619 (15)	
H17A	−0.1545	0.6122	0.4890	0.024*	
H17B	−0.2118	0.6149	0.3883	0.024*	
H17C	−0.0333	0.6808	0.4276	0.024*	
C18	0.62128 (14)	0.40534 (6)	0.40883 (6)	0.01640 (15)	
H18A	0.6235	0.3701	0.4617	0.025*	
H18B	0.7514	0.4426	0.4071	0.025*	
H18C	0.6192	0.3660	0.3588	0.025*	

### Atomic displacement parameters (Å^2^)

**Table:**

	*U*^11^	*U*^22^	*U*^33^	*U*^12^	*U*^13^	*U*^23^
O1	0.0151 (3)	0.0202 (3)	0.0153 (3)	0.0057 (2)	0.0018 (2)	0.0026 (2)
O2	0.0144 (3)	0.0191 (3)	0.0123 (3)	0.0035 (2)	0.0052 (2)	0.0029 (2)
O3	0.0157 (3)	0.0197 (3)	0.0133 (3)	0.0048 (2)	0.0024 (2)	0.0056 (2)
C1	0.0121 (3)	0.0125 (3)	0.0111 (3)	0.0000 (2)	0.0014 (2)	−0.0003 (2)
C2	0.0117 (3)	0.0116 (3)	0.0104 (3)	−0.0001 (2)	0.0013 (2)	0.0003 (2)
C3	0.0116 (3)	0.0128 (3)	0.0119 (3)	0.0004 (2)	0.0017 (2)	0.0001 (2)
C4	0.0109 (3)	0.0137 (3)	0.0111 (3)	−0.0003 (2)	0.0024 (2)	0.0000 (2)
C5	0.0122 (3)	0.0126 (3)	0.0116 (3)	0.0001 (2)	0.0005 (2)	0.0014 (2)
C6	0.0116 (3)	0.0143 (3)	0.0120 (3)	0.0013 (2)	0.0012 (2)	0.0009 (2)
C7	0.0112 (3)	0.0114 (3)	0.0110 (3)	−0.0005 (2)	0.0013 (2)	−0.0001 (2)
C8	0.0118 (3)	0.0140 (3)	0.0111 (3)	0.0013 (2)	0.0021 (2)	0.0007 (2)
C9	0.0118 (3)	0.0124 (3)	0.0115 (3)	0.0005 (2)	0.0019 (2)	0.0000 (2)
C10	0.0134 (3)	0.0130 (3)	0.0118 (3)	0.0010 (2)	0.0015 (2)	0.0003 (2)
C11	0.0136 (3)	0.0120 (3)	0.0113 (3)	−0.0004 (2)	0.0015 (2)	0.0002 (2)
C12	0.0143 (3)	0.0165 (3)	0.0133 (3)	0.0018 (3)	0.0019 (3)	0.0014 (3)
C13	0.0148 (3)	0.0193 (4)	0.0167 (4)	0.0021 (3)	0.0038 (3)	−0.0005 (3)
C14	0.0175 (4)	0.0185 (4)	0.0146 (3)	−0.0029 (3)	0.0057 (3)	−0.0015 (3)
C15	0.0186 (4)	0.0164 (3)	0.0123 (3)	−0.0030 (3)	0.0024 (3)	0.0015 (3)
C16	0.0154 (3)	0.0145 (3)	0.0126 (3)	0.0003 (2)	0.0012 (3)	0.0013 (2)
C17	0.0141 (3)	0.0189 (4)	0.0156 (3)	0.0024 (3)	0.0043 (3)	−0.0007 (3)
C18	0.0153 (3)	0.0169 (3)	0.0171 (4)	0.0032 (3)	−0.0010 (3)	0.0032 (3)

### Geometric parameters (Å, °)

**Table:**

O1—C1	1.2303 (10)	C10—C11	1.4645 (11)
O2—C4	1.3628 (10)	C10—H10A	0.9300
O2—C17	1.4366 (10)	C11—C12	1.4061 (11)
O3—C5	1.3499 (10)	C11—C16	1.4092 (11)
O3—C18	1.4338 (10)	C12—C13	1.3933 (12)
C1—C2	1.4687 (11)	C12—H12A	0.9300
C1—C9	1.5017 (11)	C13—C14	1.3941 (12)
C2—C7	1.3894 (11)	C13—H13A	0.9300
C2—C3	1.4076 (11)	C14—C15	1.3923 (13)
C3—C4	1.3805 (11)	C14—H14A	0.9300
C3—H3A	0.9300	C15—C16	1.3920 (12)
C4—C5	1.4318 (11)	C15—H15A	0.9300
C5—C6	1.3945 (11)	C16—H16A	0.9300
C6—C7	1.3977 (11)	C17—H17A	0.9600
C6—H6A	0.9300	C17—H17B	0.9600
C7—C8	1.5146 (11)	C17—H17C	0.9600
C8—C9	1.5154 (11)	C18—H18A	0.9600
C8—H8A	0.9700	C18—H18B	0.9600
C8—H8B	0.9700	C18—H18C	0.9600
C9—C10	1.3487 (11)		
			
C4—O2—C17	116.88 (6)	C9—C10—H10A	114.5
C5—O3—C18	118.07 (7)	C11—C10—H10A	114.5
O1—C1—C2	127.21 (7)	C12—C11—C16	118.04 (7)
O1—C1—C9	126.20 (7)	C12—C11—C10	124.30 (7)
C2—C1—C9	106.59 (6)	C16—C11—C10	117.65 (7)
C7—C2—C3	121.92 (7)	C13—C12—C11	120.47 (8)
C7—C2—C1	109.82 (7)	C13—C12—H12A	119.8
C3—C2—C1	128.26 (7)	C11—C12—H12A	119.8
C4—C3—C2	118.38 (7)	C12—C13—C14	120.74 (8)
C4—C3—H3A	120.8	C12—C13—H13A	119.6
C2—C3—H3A	120.8	C14—C13—H13A	119.6
O2—C4—C3	125.58 (7)	C15—C14—C13	119.50 (8)
O2—C4—C5	114.40 (7)	C15—C14—H14A	120.2
C3—C4—C5	120.02 (7)	C13—C14—H14A	120.2
O3—C5—C6	125.08 (7)	C16—C15—C14	120.02 (8)
O3—C5—C4	114.17 (7)	C16—C15—H15A	120.0
C6—C5—C4	120.75 (7)	C14—C15—H15A	120.0
C5—C6—C7	118.72 (7)	C15—C16—C11	121.21 (8)
C5—C6—H6A	120.6	C15—C16—H16A	119.4
C7—C6—H6A	120.6	C11—C16—H16A	119.4
C2—C7—C6	120.16 (7)	O2—C17—H17A	109.5
C2—C7—C8	111.85 (7)	O2—C17—H17B	109.5
C6—C7—C8	127.99 (7)	H17A—C17—H17B	109.5
C7—C8—C9	103.06 (6)	O2—C17—H17C	109.5
C7—C8—H8A	111.2	H17A—C17—H17C	109.5
C9—C8—H8A	111.2	H17B—C17—H17C	109.5
C7—C8—H8B	111.2	O3—C18—H18A	109.5
C9—C8—H8B	111.2	O3—C18—H18B	109.5
H8A—C8—H8B	109.1	H18A—C18—H18B	109.5
C10—C9—C1	119.86 (7)	O3—C18—H18C	109.5
C10—C9—C8	131.47 (7)	H18A—C18—H18C	109.5
C1—C9—C8	108.67 (6)	H18B—C18—H18C	109.5
C9—C10—C11	130.91 (7)		
			
O1—C1—C2—C7	179.73 (8)	C5—C6—C7—C2	0.24 (11)
C9—C1—C2—C7	−0.12 (8)	C5—C6—C7—C8	−178.92 (7)
O1—C1—C2—C3	0.09 (13)	C2—C7—C8—C9	−1.30 (8)
C9—C1—C2—C3	−179.76 (7)	C6—C7—C8—C9	177.91 (8)
C7—C2—C3—C4	−0.86 (11)	O1—C1—C9—C10	−0.86 (13)
C1—C2—C3—C4	178.74 (7)	C2—C1—C9—C10	179.00 (7)
C17—O2—C4—C3	−14.67 (11)	O1—C1—C9—C8	179.44 (8)
C17—O2—C4—C5	165.99 (7)	C2—C1—C9—C8	−0.71 (8)
C2—C3—C4—O2	179.62 (7)	C7—C8—C9—C10	−178.48 (8)
C2—C3—C4—C5	−1.07 (11)	C7—C8—C9—C1	1.18 (8)
C18—O3—C5—C6	−1.11 (12)	C1—C9—C10—C11	−179.66 (8)
C18—O3—C5—C4	179.19 (7)	C8—C9—C10—C11	−0.03 (15)
O2—C4—C5—O3	1.72 (10)	C9—C10—C11—C12	−1.40 (14)
C3—C4—C5—O3	−177.66 (7)	C9—C10—C11—C16	178.97 (8)
O2—C4—C5—C6	−178.00 (7)	C16—C11—C12—C13	−0.44 (12)
C3—C4—C5—C6	2.62 (12)	C10—C11—C12—C13	179.93 (8)
O3—C5—C6—C7	178.15 (7)	C11—C12—C13—C14	0.46 (13)
C4—C5—C6—C7	−2.16 (12)	C12—C13—C14—C15	0.04 (13)
C3—C2—C7—C6	1.31 (11)	C13—C14—C15—C16	−0.54 (13)
C1—C2—C7—C6	−178.36 (7)	C14—C15—C16—C11	0.55 (12)
C3—C2—C7—C8	−179.41 (7)	C12—C11—C16—C15	−0.06 (12)
C1—C2—C7—C8	0.92 (9)	C10—C11—C16—C15	179.60 (7)

### Hydrogen-bond geometry (Å, °)

**Table:**

Cg1 is the centroid of C2–C7 benzene ring.

**Table:**

*D*—H···*A*	*D*—H	H···*A*	*D*···*A*	*D*—H···*A*
C17—H17A···O3^i^	0.96	2.53	3.4107 (11)	152
C18—H18B···O2^ii^	0.96	2.58	3.5320 (11)	173
C16—H16A···Cg1^iii^	0.93	2.99	3.7224 (9)	137

Symmetry codes: (i) −*x*, −*y*+1, −*z*+1; (ii) *x*+1, *y*, *z*; (iii) *x*, −*y*+1/2, *z*−3/2.
